# ePROs in the follow-up of cancer patients treated with immune checkpoint inhibitors: a retrospective study

**DOI:** 10.1007/s00432-018-02835-6

**Published:** 2019-01-21

**Authors:** Sanna Iivanainen, Tuomo Alanko, Katriina Peltola, Teemu Konkola, Jussi Ekström, Henri Virtanen, Jussi P. Koivunen

**Affiliations:** 1 Department of Oncology and Radiotherapy, Oulu University Hospital (OYS), MRC Oulu, P.B. 22, 90029 Oulu, Finland; 2 Docrates Cancer Center, Helsinki, Finland; 3 Kaiku Health Oy, Helsinki, Finland

**Keywords:** Patient-reported outcomes, Immune checkpoint inhibitor therapy, Symptoms, Real-world data

## Abstract

**Purpose:**

Patient-reported outcome (PRO) follow-up has been shown to improve quality of life (QoL) and survival of cancer patients receiving chemotherapy. Kaiku Health application is a web-based electronic PRO (ePRO) tool which is designed for follow-up of cancer patients receiving immune checkpoint inhibitors (ICI). Purpose of the current study is to investigate whether symptoms collected by Kaiku Health ePRO tool on cancer patients receiving immune checkpoint inhibitors (ICI) follows to symptoms reported in clinical trials and whether coupling of specific symptoms does occur.

**Methods:**

We retrospectively collected data on symptom timing and severity, and QoL of patients followed with Kaiku Health IO module in two Finnish cancer centers between 2017 and 2018. Kaiku Health IO module consists of 18 adaptive questions, which assess the presence and severity of symptoms. Patients were requested (via e-mail) to fill online symptom questionnaires with 3–7 day interval and QoL questionnaires (QLQ-C30) with 1–2 month interval.

**Results:**

The IO module was used to follow 37 patients who had filled in total 559 symptom questionnaires. There was good adherence to ePRO follow-up with a median of 11 questionnaires filled per patient. The reported symptoms and their severity follow closely what has been seen in clinical trials investigating ICIs. Correlation analysis of the symptoms showed the strongest positive correlations between itching and rash; nausea and vomiting, decreased appetite, or stomach pain; cough and shortness of breath.

**Conclusions:**

The results of the current study suggest that real-world symptom data collected through the ePRO application on cancer patients receiving ICI therapy aligns with the data from clinical trials. Correlations between different symptoms occur, which might reflect therapeutic efficiency, side effects, or tumor progression. These correlations should be further investigated with data coupled to clinical outcomes.

## Introduction

Cancer patients suffer from a variety of symptoms derived from the malignancy itself, whereas some arise as side effects of the given cancer treatments. Many symptoms are left unnoticed due to factors such as timely discontinuity between prescheduled health care appointments, individual disease history, and inadequate patient coherence (Reilly et al. 2013; Henry et al. 2008; Laugsand et al. 2010; Basch et al. 2009; Gilbert et al. 2012; Valderas et al. 2008; Velikova et al. 2010). In general, worsening of symptoms indicates cancer progression or severe side effects of the treatment and is linked to poorer cancer survival (Trajkovic-Vidakovic et al. 2012). Scheduled electronic patient-reported outcomes (ePROs) enable timely and continuous collection of symptoms in cost-effective manner (Jensen et al. 2014; Kotronoulas et al. 2014; Bennett et al. 2012; Cleeland et al. 2011; Holch et al. 2017; Mullen et al. 2004; Pakhomov et al. 2008). If ePROs are linked to an urgency algorithm, they offer a chance for prompt reaction to important medical events. Web-based applications coupled to urgency algorithm have been developed to monitor cancer patients, and currently, the most convincing data exist on patients receiving chemotherapy or undergoing follow-up for lung cancer (Basch et al. 2016; Denis et al. 2017). ePROs have been shown to improve quality of life (QoL), decrease emergency clinic visits, and improve Eastern Cooperative Oncology Group (ECOG) performance status and the number of patients receiving active cancer treatments at disease progression (Basch et al. 2016; Denis et al. 2017; Velikova et al. 2004). Furthermore, use of ePROs in patient monitoring has shown impressive improvements in overall survival compared to standard follow-up (Basch et al. 2017; Denis et al. 2017a, b). Increasing use of smartphones and apps in the general population supports the idea of collection of individual health data based on such communication channels (Benze et al. 2017). Nevertheless, web-based applications can be designed as scalable to take into account different user interfaces.

In the past 5 years, there has been a huge development in cancer immunotherapy with introduction of immune checkpoint inhibitor therapies such as PD-(L)1 and CTLA-4 antibodies (Brahmer et al. 2015; Wolchok et al. 2017; Borghaei et al. 2015; Motzer et al. 2015; Bellmunt et al. 2017; Robert et al. 2015a, b; Herbst et al. 2016; Rittmeyer et al. 2017; Reck et al. 2016). The immune checkpoint inhibitors act through inhibition of T-cell blocking which results in T-cell-mediated cancer cell death. The side effects of immune checkpoint inhibitors resemble autoimmune disease. The most common ones are rash, endocrine toxicity, GI toxicity, hepatitis, and pneumonitis. Even life-threatening side effects can occur, but they can, in most cases, be managed with early detection, delaying or stopping of the immuno-oncological (IO) therapy and initiation of immunosuppressive medication, most commonly corticosteroids (Spain et al. 2016; Puzanov et al. 2017; Haanen et al. 2017; Wang et al. 2018). Timing of side effects differs from traditional cancer therapy and they can occur from months to years after therapy initiation or after discontinuation of the therapy (Li et al. 2017; McDermott et al. 2015; Weber et al. 2017). Therefore, long-term follow-up of patients even after therapy discontinuation is warranted.

There are no published works investigating ePRO follow-up approach on cancer patients treated with ICIs. Current study investigates symptoms collected by ePRO tool on cancer patients receiving ICIs and their correlation to data presented on clinical trials and coupling of the reported symptoms. The study hypothesis are that ePRO collection of symptoms would be similar or higher compared to clinical trials and certain symptoms co-occur.

## Methods

### Patients

All the study patients included had cancer being treated with IO therapy at Docrates Cancer Center (Helsinki, Finland) and Oulu University Hospital (Oulu, Finland) in outpatient setting 4/2017–9/2018 and they were followed with Kaiku ePRO module and had at least one symptom questionnaire filled. Data on symptom and QoL questionnaires were retrospectively collected from data registry of Kaiku Health at 9/2018 of all the patients filling the inclusion criteria. Of the clinical variables, the registry included only age and sex of the patients but no additional clinical variables. Data collection was done under permits from Kaiku Health, Docrates Cancer Center, and Oulu University Hospital Ethics Committee (9/2017).

### ePRO follow-up

Kaiku Health ePRO tool is a web-based solution scaled to be used fluently in smartphones and home computers. Kaiku Health IO module developed by Kaiku Health consists of 18 questions. The symptoms selected for the Kaiku Health symptom-tracking tool for cancer immunotherapy are based on the most common adverse events that have occurred during clinical trials of anti-PD-1, anti-PD-L1, and anti-CTLA4 monotherapies. The symptoms tracked by the instrument are potential signs and symptoms of immune-related adverse events. The symptom selection is based on the reported publications of following clinical trials: CheckMate 017, CheckMate 026, CheckMate 057, CheckMate 066, CheckMate 067, KEYNOTE-010, and OAK. FDA labels for Nivolumab, Pembrolizumab, and Atezolizumab were also used in the symptom selection for the instrument. The questions for each symptom in the instrument were developed based on NCI-CTCAE v.4.03 register by converting the description of gradings into a patient-friendly language. Any criteria that are impossible for patients to report have been excluded from the available questions. Developing the symptom questionnaire in this manner has enabled the self-reporting of patients and development of an algorithm that provides an assessment and an approximation of the severity of each symptom.

Questions asses the presence of blood in stool, blood in urine, blurred vision, chest pain, cough, decreased appetite, diarrhea, dizziness, fatique, fever, headache, itching, nausea, other symptoms, pain in joints, rash, shortness of breath, stomach pain, and vomiting. Besides recording a presence of a symptom, the application has a severity algorithm that grades the symptom according to NCI-CTAE v. 4.03 protocol. Furthermore, the application has a feature of an urgency algorithm that sends out alerts to the care unit when the patient reports predefined severe or altering symptoms (limits set by care unit). This feature could be activated by the care unit, but this was used only in part of the study patients and the data on alerts was excluded from the analysis. QoL was captured with electronic QLQ-C30-questionnaire included in the Kaiku ePRO module.

## Results

### Patient cohort and engagement to ePRO follow-up

**Fig. 1:**
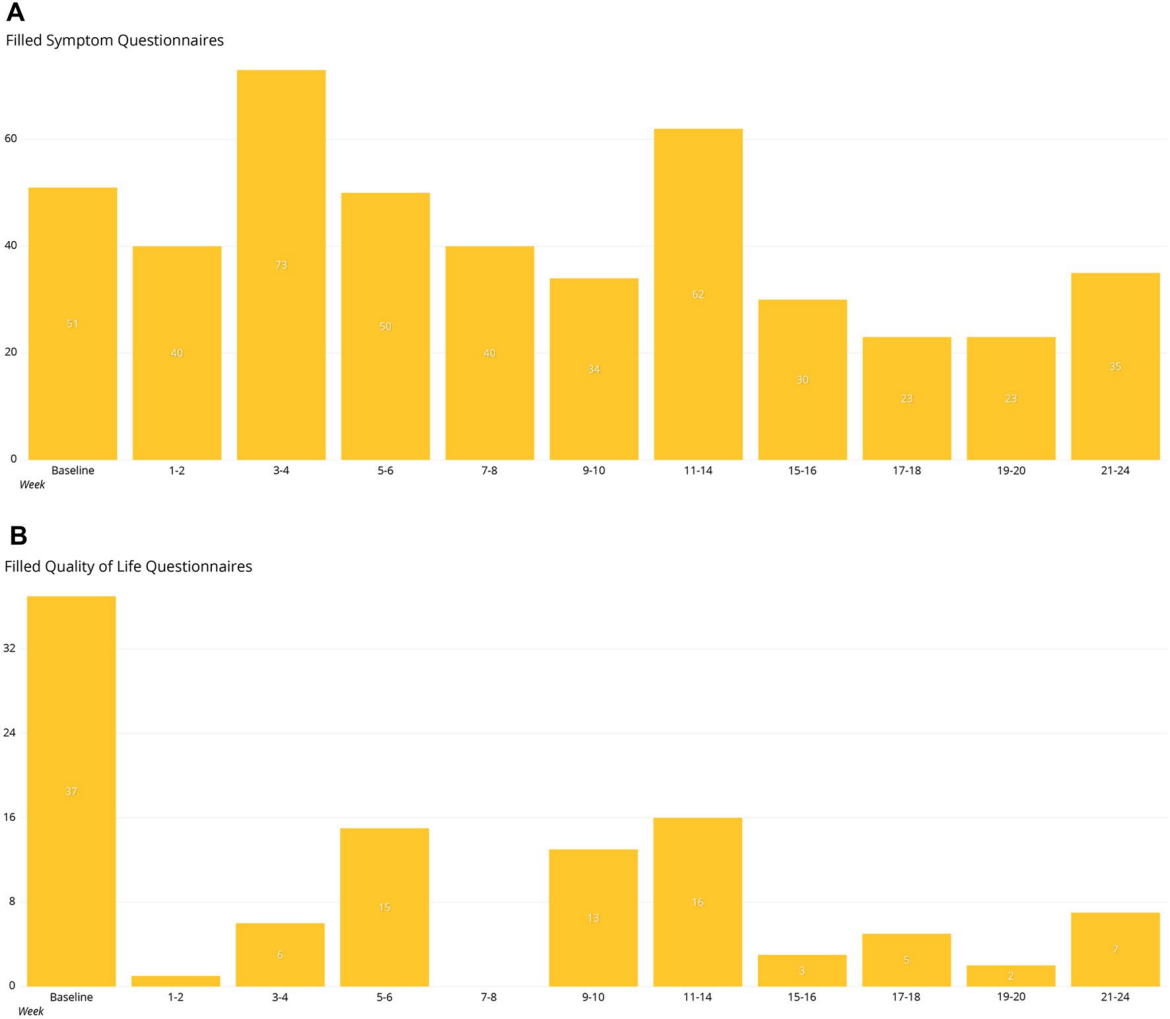
Number of filled questionnaires in timely relation to the baseline **a** symptom questionnaires, **b** quality-of-life questionnaires

A total of 37 patients with median age of 61 were included in the study. 24 (64.9%) of the patients were male. The subjects filled 559 questionnaires focusing on known immunologically related adverse events (irAEs) and 133 QoL questionnaires. There was good compliance to ePRO surveillance up to 24 weeks from baseline. The answering rate to symptom questionnaires was highest at 3–4 weeks (the number of symptom questionnaires filled *n* = 73), 11–14 weeks (*n* = 62), and baseline (*n* = 51) (Fig. 1a). The median number of filled symptom questionnaires was 11 per patient (CI 1–47; SD 12.3). All the patients included had initial QoL questionnaires filled, but the answering rate was much lower at later time points analyzed (Fig. 1b). Median number of filled QoL questionnaires was two (CI 1–18; SD 3.47), and the answering rate was highest at 11–14 weeks.

At the time of analysis, most patients (*n* = 28) had > 12 weeks from the first filled symptom questionnaire and 19 of 28 (67.9%) patients had continued the symptom reporting for > 12 weeks, suggesting good adherence to ePRO follow-up. Of the 28 patients, one (3.6%) had the highest reported symptoms at severity grade 0, seven (25%) at grade 2, and 20 patients (71.4%) at grade ≥ 3. According to reported symptom severity, patients with grade 0 had on average 0.1 questionnaires filled per week, grade 2 patients 0.65 questionnaires/week, and grade ≥ 3 patients 0.66 questionnaires/week.

### Symptoms and QoL during ePRO follow-up

**Fig. 2:**
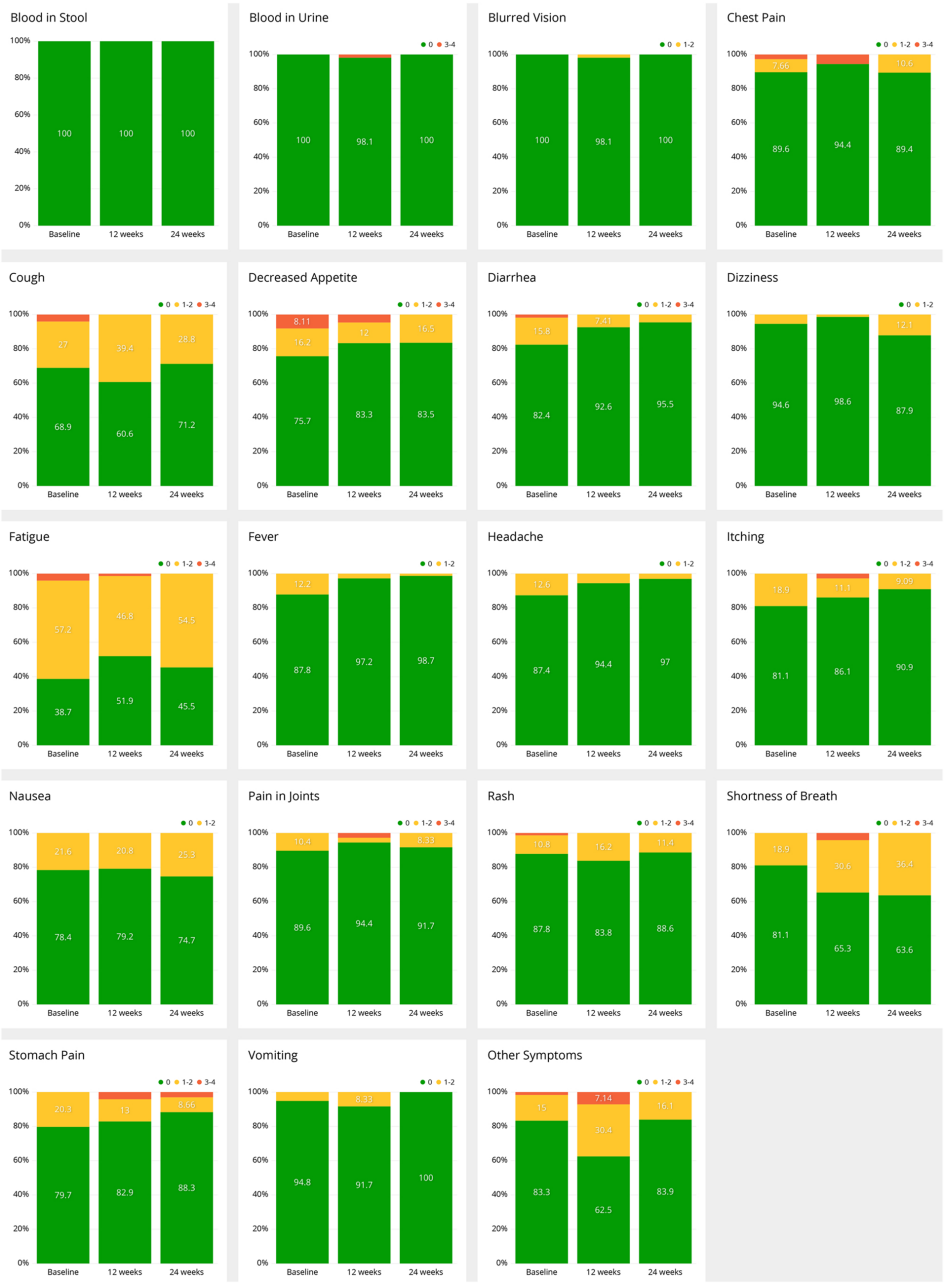
Development of specific symptoms at baseline, 12 and 24 weeks according to NCI-CTCAE grading (0, 1–2, 3–4) Grade 0 is marked with green, grade 1–2 yellow, and grade 3–4 red

Email remainders to patients of symptom questionnaires to be filled were sent out initially and, thereafter, at 3–7 day frequency. The reported symptoms were categorized by severity algorithm of the application (grade 0–4) and grouped to timely to baseline, 12 and 24 weeks. Symptoms and their grading in all the filled questionnaires are presented in Table 1. The most common reported grade 1–2 symptoms were fatigue (47%), shortness of breath (31%), and cough (29%). Of the grade 3–4 symptoms, decreased appetite (5%), other symptoms (5%), and chest pain (3%) were the most frequent. The development of symptoms and their severity from baseline to 12 and 24 weeks is presented in Fig. 2. Since urgency algorithm was activated only on some of patients, data on the urgency algorithm alerts was excluded from the current analysis.

**Table 1:** Distribution of severity of the reported symptoms according to all the answered (n = 559) symptom questionnaires

Symptom	Grade 0 (%)	Grades 1–2 (%)	Grades 3–4 (%)
Blood in stool	100	0	0
Blood in urine	99	1	1
Blurred vision	98	1	0
Chest pain	92	6	3
Cough	69	29	2
Decreased appetite	82	13	5
Diarrhea	95	5	1
Dizziness	93	6	1
Fatique	50	47	3
Fever	96	4	0
Headache	93	7	0
Itching	86	12	2
Nausea	84	15	1
Other symptoms	71	24	5
Pain in joints	85	13	2
Rash	87	13	1
Shortness of breath	67	31	2
Stomach pain	90	9	1
Vomiting	97	3	0

**Fig. 3:**
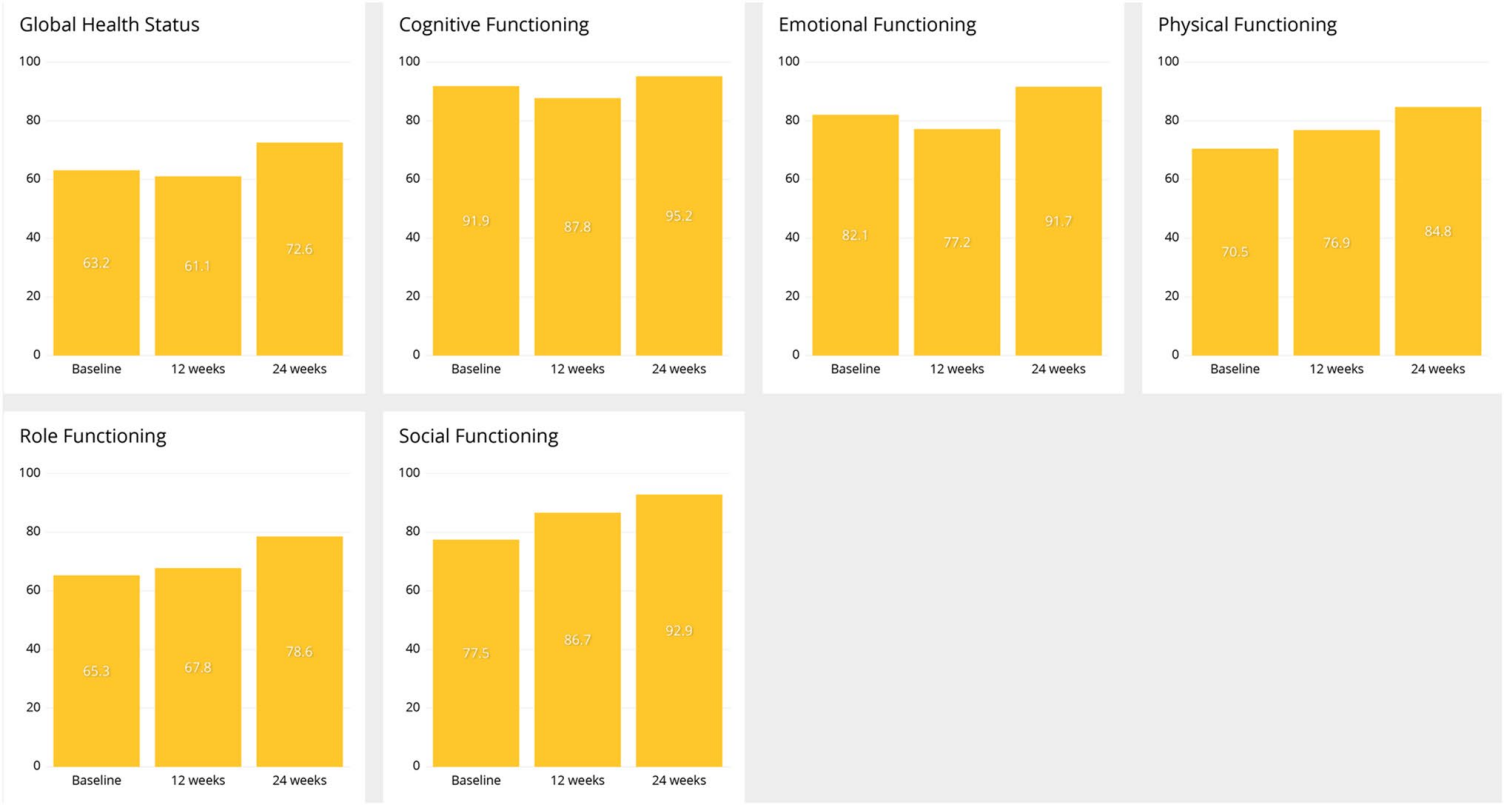
Development of QoL according QLQ-C30 global health status and functioning scales at baseline, 12 and 24 weeks

Email remainders to patients of QoL questionnaires to be filled were sent out initially and, thereafter, at 1–2 months frequency. QLQ-C30 scales are presented in Fig. 3. In general, there was tendency for improvement in all the scales from baseline to 12 and 24 weeks.

### Symptom and QoL correlations

**Fig. 4:**
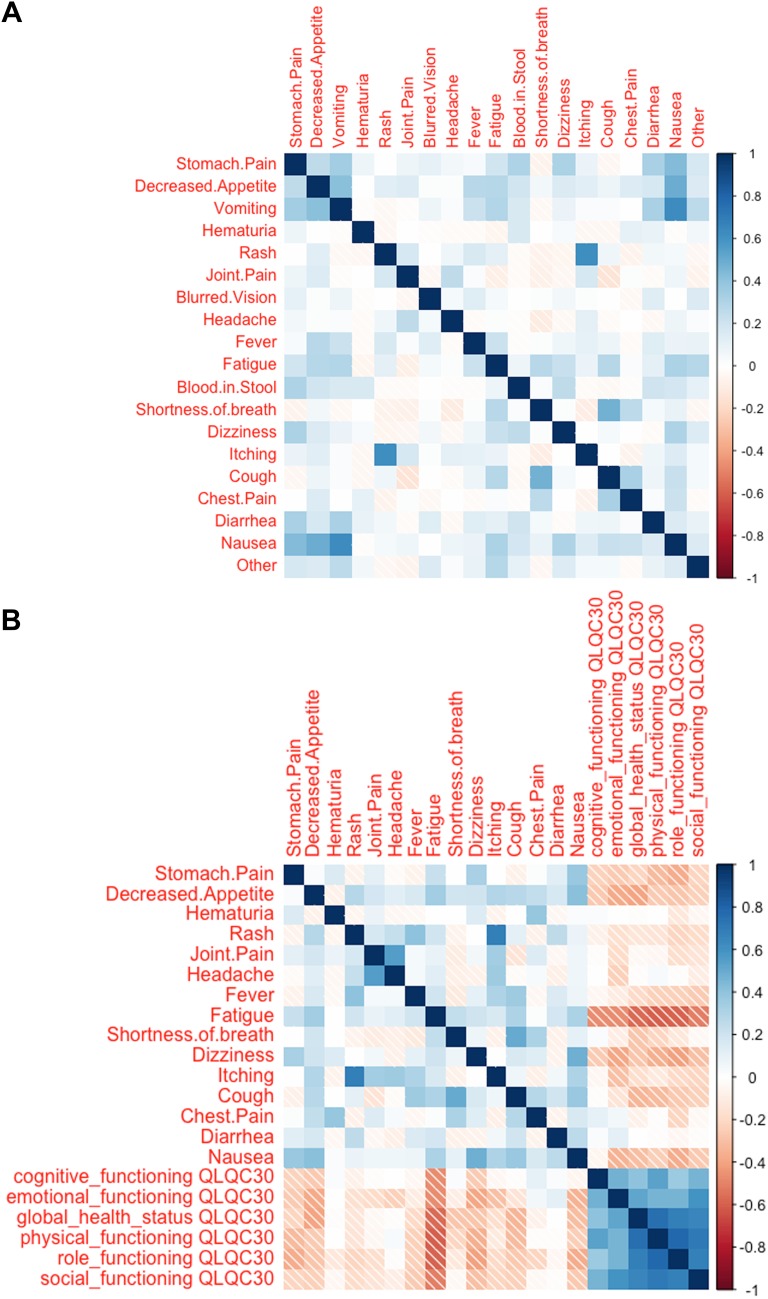
Correlation analysis using heat maps **a** correlation between reported symptoms, **b** correlation between symptoms and QoL by QLQ-C30 global health status and functioning scales The intensity of the color signifies the level of correlation; red negative, blue positive correlation. Strong correlation ratio is defined > 0.5 or < − 0.5; intermediate 0.5–0.3 or − 0.5 to − 0.3; weak 0.3–0.1 or − 0.3 to − 0.1; very weak 0.1 to − 0.1

Correlations of different patient-reported symptoms were analyzed using heat maps. The strongest positive correlations were seen between itching and rash, and between nausea and vomiting. Furthermore, positive correlations were seen between nausea, decreased appetite, and stomach pain; and cough and shortness of breath. Analyzes did not show high level of negative correlations between individual symptoms. Interestingly, however, negative correlations were seen between certain groups of symptoms. Rash, itching, joint pain, and diarrhea negatively correlated with cough, shortness of breath, and chest pain (Fig. 4a).

The same method was also applied to correlation analysis between QoL scales (QLQ-C30) and patient-reported symptoms. According to the results, of the reported symptoms, lower QoL scale and lower global health status had strongest correlation with fatigue, decreased appetite, nausea, and dizziness (Fig. 4b).

## Discussion

The toxicity spectrum of immune checkpoint inhibitor therapies is wide and inadequately characterized (Le Burel et al. 2017; Pillai et al. 2018). Timing of AEs with traditional chemotherapies and targeted therapies is strongly connected to the initiation of the medication, while irAEs typically occur later in the treatment course or even after discontinuing the therapy. Furthermore, even though some irAEs are more common than others, the spectrum of potential AEs is wider compared to traditional cancer medications. Early detection of irAEs is suggested to improve their management and decrease the need for hospitalization (Champiat et al. 2016; Michot et al. 2016). To our knowledge, the current study is the first investigating ePROs in the follow-up of cancer patients receiving immune checkpoint inhibitor therapies. Our study provides evidence that this approach is feasible based on the good patient adherence and strong correlation of AE spectrum to the previous clinical trials.

The answering rate to QoL questionnaires was much lower (median 2) compared to symptom reporting rate. This is probably due to the lower frequency of QoL-questionnaire scheduling (every 1–2 months) compared to symptom questionnaires (every 3 days to weekly). Furthermore, compliance to QoL questionnaires might be lower because of the more complicated nature of the questionnaires making them less appealing to the patient.

Patient-reported real-world data (RWD) on immune checkpoint inhibitor related side effects is limited. The variety and severity of patient-reported symptoms in our study seem to follow closely to what has been seen in the clinical trials investigating ICIs (Brahmer et al. 2015; Robert et al. 2015; Wolchok et al. 2017; Borghaei et al. 2015; Herbst et al. 2016; Rittmeyer et al. 2017; Reck et al. 2016; Schachter et al. 2017). For example, respiratory events like cough and dyspnea have been documented in up to 20–40% of patients receiving anti-PD-(L)1-therapies with grade 3–4 cough in 2–9% (Haanen et al. 2017) which is closely congruent with our data (29/31% and 2/2%). Furthermore, frequencies of arthralgia and rash (both 13%) are very similar to clinical studies (both 15%) (Michot et al. 2016; Suarez-Almazor et al. 2017).

The symptom correlation analysis revealed coupling of certain symptoms with positive and negative correlations. Strong positive correlations were seen between predicted irAEs like rash and itching, and on the other hand, pulmonary symptoms (cough and shortness of breath). Interestingly, rash, itching and joint pain, typical irAEs, had negative correlations with cough, shortness of breath and chest pain which often are related to disease progression in the context of lung cancer or lung metastases (Haanen 2017). Our finding of negative correlation between well-known irAEs and disease progression-related symptoms supports the hypothesis that incidence of irAEs with ICI therapies is suggestive of potential clinical benefit to the patient (Fujii et al. 2018). Data of the current study did not include clinical outcomes excluding age and sex, and therefore, symptom correlations are hypothesis generating only. This should be further investigated with comprehensive, prospective study linking symptoms to clinical outcomes.

The correlation of patient-reported symptoms to QoL (global health status and functioning scales) showed in general highly negative correlations excluding joint pain, headache, shortness of breath, chest pain and diarrhea. This suggests that clinical decision-making aiming to improve patients’ well-being based on merely QoL-reporting is difficult. Our findings support the idea that the clinical value of follow-up of cancer patients is increased when individualized symptom analysis is combined to standard measurements (Velikova et al. 2008) .

In summary, our results show that ePRO follow-up of cancer patients treated with immune checkpoint inhibitors is feasible. The symptom variety, incidence, and grading collected with ePRO questionnaire from real-world patients mimics what has been reported in anti-PD-(L)1-trials making our results clinically convincing. The correlation analysis showed a negative correlation between common irAEs and symptoms suggesting disease progression which should be further investigated with data linked to clinical outcomes.
